# Feasibility of using community pharmacy sales of over-the-counter respiratory products as a syndromic surveillance indicator: a pilot study from Sofia, Bulgaria

**DOI:** 10.3389/fpubh.2026.1886507

**Published:** 2026-09-24

**Authors:** Zhivka Getsova, Vanya Rangelova, Venelin Sapunarov, Ivo Georgiev, Kalina Petkova, Valeri Tzekov, Galin Kamenov

**Affiliations:** 1 Epidemiology Department, National Centre of Infectious and Parasitic Diseases (NCIPD), Sofia, Bulgaria; 2 Center of Competence ImmunoPathogen, Sofia, Bulgaria; 3 Department of Epidemiology and Disaster Medicine, Faculty of Public Health, Medical University of Plovdiv, Plovdiv, Bulgaria; 4 Regional Pharmaceutical College - Sofia Capital City, Sofia, Bulgaria; 5 Bulgarian Society for Innovative Medicine, Plovdiv, Bulgaria; 6 Second Department of Internal Diseases, Section of Nephrology, Medical University of Plovdiv, Plovdiv, Bulgaria; 7 First Dialysis Services Bulgaria—Dialysis Center, Plovdiv, Bulgaria

**Keywords:** early warning, pharmaceutical products, pharmacy network, respiratory symptoms, surveillance, syndromic surveillance

## Abstract

**Introduction:**

Data on sales of pharmaceutical products (SPP) for symptomatic treatment of acute respiratory diseases can be a reliable indicator providing initial information before official surveillance systems. This pilot study aimed to develop a framework for collecting community pharmacy sales data and assess the feasibility of using these data as a complementary indicator of respiratory infection activity.

**Methods:**

Products were selected through observation of prime-time television advertisements during February–March 2025, expert review, and a survey of 97 community pharmacists. Retrospective monthly sales data for selected product categories were obtained from two community pharmacies in Sofia for October 2024–April 2025. Sales were analysed using descriptive statistics, the Friedman test, and post-hoc Wilcoxon signed-rank tests with Bonferroni correction.

**Results:**

Mean SPP vary from 121.06 units for antivirals to 685.96 units for antipyretics. Antivirals showed the greatest relative monthly variability (*R* = 216.4, CV = 64.78), followed by food supplements (*R* = 160.16, CV = 33.23). Sales of products for sore throat were comparatively stable (*R* = 103, CV = 18.62) with limited temporal variation during the observation period. Antipyretic and antiviral sales reached their highest levels in January 2025, accounting for 19.14 and 32.00% of their respective total sales during the observation period. The January increase temporally coincided with the peak in reported acute respiratory infections in Sofia during week 4 of 2025. However, pairwise comparisons between January and the other months were not statistically significant after Bonferroni correction.

**Conclusion:**

Pharmacy sales data showed preliminary potential for monitoring local respiratory infection trends; however, larger prospective multicenter studies using standardized product classifications and higher-frequency sales data are needed to evaluate their contribution to complementary respiratory syndromic surveillance.

## 1 Introduction

The International Health Regulations (IHR 2005) require countries to develop surveillance systems capable of the early detection and reporting of public health threats, including respiratory infectious diseases, in order to ensure timely outbreak response and containment (1, 2). Respiratory pathogens are of particular concern because of their epidemic potential and the speed with which they can spread within communities (3). Nevertheless, many countries still experience limitations in achieving the surveillance capacities required under the IHR, highlighting the need for complementary approaches that can support earlier situational awareness (2). Among these, syndromic surveillance has become an increasingly important strategy, as it relies on symptoms and healthcare-seeking behaviour rather than laboratory-confirmed diagnoses to detect unusual health events at an early stage (4–7).

Syndromic surveillance systems integrate diverse data sources, depending on their availability within the local healthcare and social context, to support early detection, situational awareness, and public health decision-making (8). These systems combine clinical data, such as emergency department visits, with behavioural data, including over-the-counter medication sales, participatory online surveys, and school or workplace absenteeism (9–13). Multiple data streams offer complementary surveillance capabilities, as different sources detect different outbreak types with varying efficacy and timeliness. For instance, emergency department data may be most sensitive for severe illnesses affecting people over 65 populations, while school-based absenteeism can detect influenza activity 1–4 weeks before peak disease onset.

Among these alternative data sources, sales of pharmaceutical products (SPP) for symptomatic treatment of respiratory infections have attracted considerable interest as a potential early surveillance indicator (14, 15). Previous studies have demonstrated correlations between over-the-counter (OTC) medication sales and respiratory disease activity, with reported correlation coefficients ranging from 0.36 to 0.80 depending on the product category (16). In China, combined monitoring of antitussive, expectorant, and influenza medications predicted respiratory disease epidemics with an accuracy of 83.3% (16), while studies from the United States found strong correlations between pharmacy prescription sales, Google Flu Trends, and CDC influenza surveillance data (15). Because individuals frequently self-manage respiratory symptoms before seeking medical care, increases in the sales of antipyretics, cough remedies, nasal preparations, and other symptomatic treatments may reflect community transmission before routine surveillance systems fully capture disease activity (17–23).

Research demonstrates that shifts in medication demand can precede signals captured through traditional clinical surveillance, although the extent of this lead varies by disease type. For instance, antiviral prescription dispensing data often increase in advance of laboratory-confirmed influenza reports and provide near-real-time intelligence for characterizing local influenza activity (23, 24). Elevated sales of treatments for diarrheal illness have been observed well in advance of confirmed outbreaks, whereas signals linked to respiratory infections tend to occur only a few days before detection in emergency care settings (23, 25). OTC pharmaceutical sales have been shown to explain 36% of variance in patient volume at urgent care facilities, with cough and cold medication sales significantly associated with patient volume during flu season with a lag of two days (24).

Furthermore, pharmacy data can closely track epidemic curves observed in traditional surveillance systems while being available several days to weeks before official reporting (26). Machine learning models integrating non-prescription medication sales data significantly outperform models using only traditional variables (socio-demographics and weather), with accuracy gains highest during periods of maximum public health risk (27). Despite providing earlier indications, pharmacy-derived data are generally less sensitive than clinical indicators, as their performance depends on symptom severity and patterns of self-medication (28).

The utility of pharmaceutical sales surveillance varies considerably across settings and populations. Studies from the 2009 H1N1 pandemic in Great Britain found no significant national-level correlation between retail sales of symptom remedies and influenza cases, though significant positive correlations emerged at regional scales, particularly for children’s symptomatic remedies (35.6% of regions) and for thermometers and antiviral hand products (29). Evidence from multiple countries shows mixed results, with some studies demonstrating strong correlations between OTC drug sales and influenza activity while others found no correlation, suggesting that local healthcare access patterns, regulatory environments, and cultural health-seeking behaviors significantly influence surveillance performance (4, 30).

Consequently, the applicability of pharmacy sales surveillance should be assessed within individual healthcare settings before broader implementation. Bulgaria has a distinct healthcare system, patterns of OTC medicine use, pharmacy distribution, and healthcare-seeking behaviour that differ from those of countries where pharmacy-based surveillance has previously been evaluated (31). Therefore, local evidence is required to determine whether pharmacy sales data can feasibly contribute to complementary respiratory syndromic surveillance.

The present pilot study aimed to propose a framework for collecting community pharmacy sales data and to evaluate the feasibility of using these data as a complementary indicator for respiratory syndromic surveillance.

## 2 Materials and methods

### 2.1 Study design and product selection

A two-step selection approach was used to identify OTC pharmaceutical products relevant for respiratory infection monitoring. First, epidemiologists conducted a systematic observation of prime-time commercial breaks on Bulgarian television during February–March 2025 to identify advertised products for respiratory symptoms, including fever, sore throat, nasal congestion, rhinorrhea, and cough, or those mentioning viral respiratory pathogens such as influenza or other respiratory viruses. A provisional list of OTC medicines and supplements was compiled and categorized into antipyretics, throat-pain relief products, nasal congestion/rhinorrhea remedies, cough relief products, antivirals, and food supplements (32, 33).

In the second step, pharmacy experts reviewed the list, after which a survey was distributed to community pharmacists through the Regional Pharmaceutical College of Sofia. Ninety-seven pharmacists (convenience sample) indicated which of the TV-advertised products they routinely recommend and could add any additional products they considered relevant. This two-step design aligns with established evidence on determinants of OTC consumer choice.

### 2.2 Sales data collection

Following product selection, retrospective monthly sales data were obtained from two community pharmacies located in Sofia for the period October 2024 through April 2025, corresponding to one respiratory infection season.

Pharmacy 1 provided aggregated monthly sales data for antipyretics, throat-pain products, nasal congestion/rhinorrhoea remedies, antivirals, and food supplements. Pharmacy 2 contributed monthly data for antipyretics, antivirals, and food supplements.

To avoid commercial bias and preserve confidentiality, sales were analysed exclusively at the product-category level, and no information identifying individual commercial brands or pharmacies was reported.

Because this investigation was designed as a pilot feasibility study, only two pharmacies participated. The limited number of pharmacies was considered sufficient to evaluate the practicality of data collection and explore temporal sales patterns but does not permit conclusions regarding representativeness at either the city or national level.

### 2.3 Statistical analysis

Monthly sales were analysed descriptively for each surveillance category. Mean values, standard deviations (SD), coefficients of variation (CV), and ranges (R) were calculated to describe temporal variability. To facilitate comparison across product categories with different absolute sales volumes, monthly sales were additionally expressed as percentages of total category-specific sales over the observation period.

Because the dataset consisted of repeated monthly observations with a small sample size and did not satisfy assumptions for parametric analysis, non-parametric statistical methods were applied. Overall differences in monthly sales distributions were evaluated using the Friedman test. Where appropriate, pairwise comparisons between January and the remaining months were performed using Wilcoxon signed-rank tests with Bonferroni correction for multiple testing (adjusted significance level *α* = 0.008).

Given the limited number of observations and monthly aggregation of the data, no formal time-series, lag, or cross-correlation analyses were performed. Consequently, the study was not designed to evaluate predictive performance or estimate whether pharmacy sales preceded routine epidemiological surveillance.

Statistical analyses were performed using IBM SPSS Statistics (Version 25), and statistical significance was defined as *p* < 0.05 unless otherwise specified.

## 3 Results

### 3.1 Distribution of sales by product category

Monthly SPP counts reported by the two pharmacies vary from 22 sales of antiviral products in April 2025 to 503 sales of antipyretics in January 2025. Antipyretics represented the largest proportion of monitored sales in both pharmacies, accounting for 35.74% of all included product sales in Pharmacy 1 and 74.45% in Pharmacy 2. In Pharmacy 1, nasal congestion and rhinorrhoea remedies were the second most frequently sold category, accounting for 28.29% of monitored sales (Figures 1, 2).

**Figure 1:**
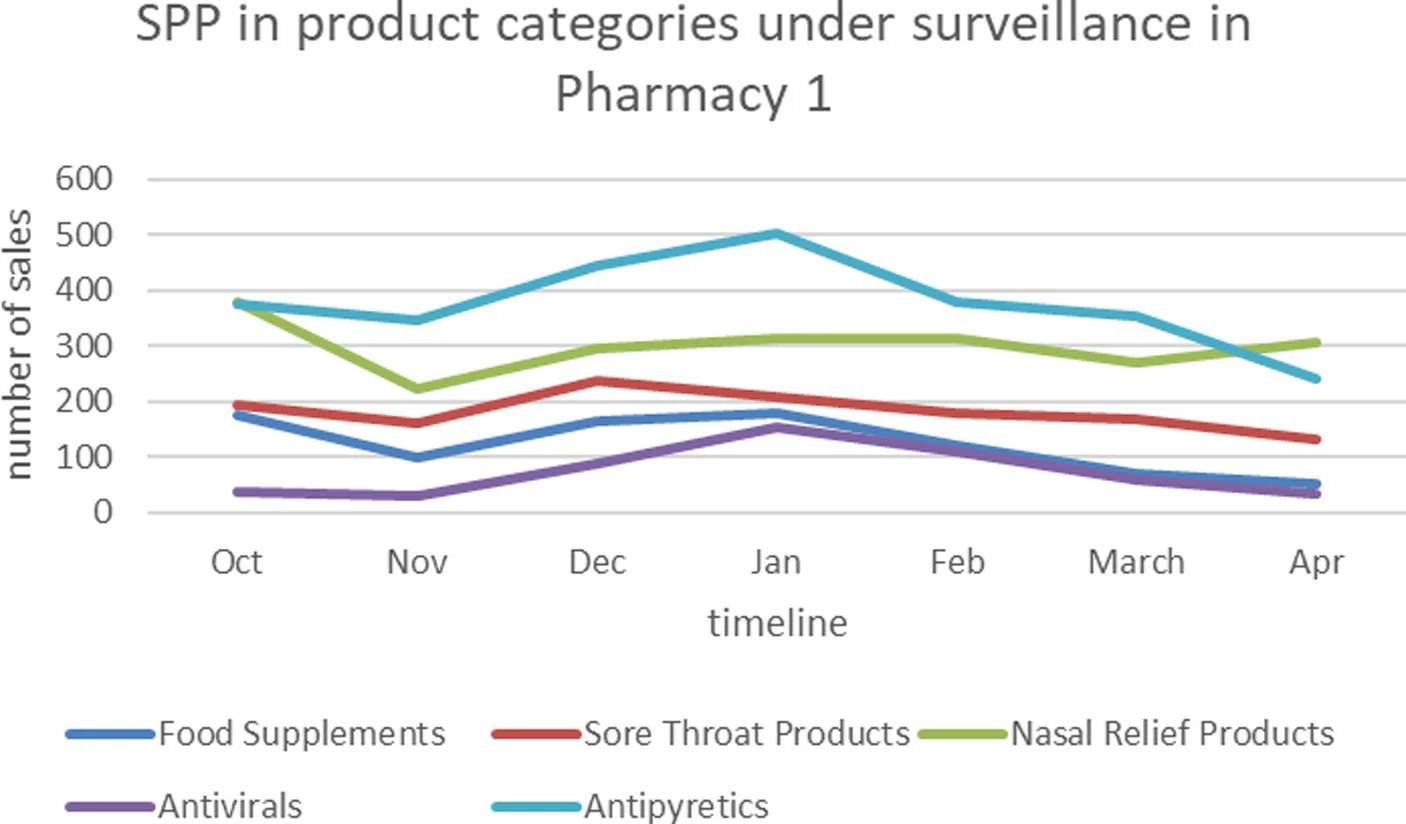
Distribution of sales across the monitored product categories in Pharmacy 1, October 2024–April 2025.

**Figure 2:**
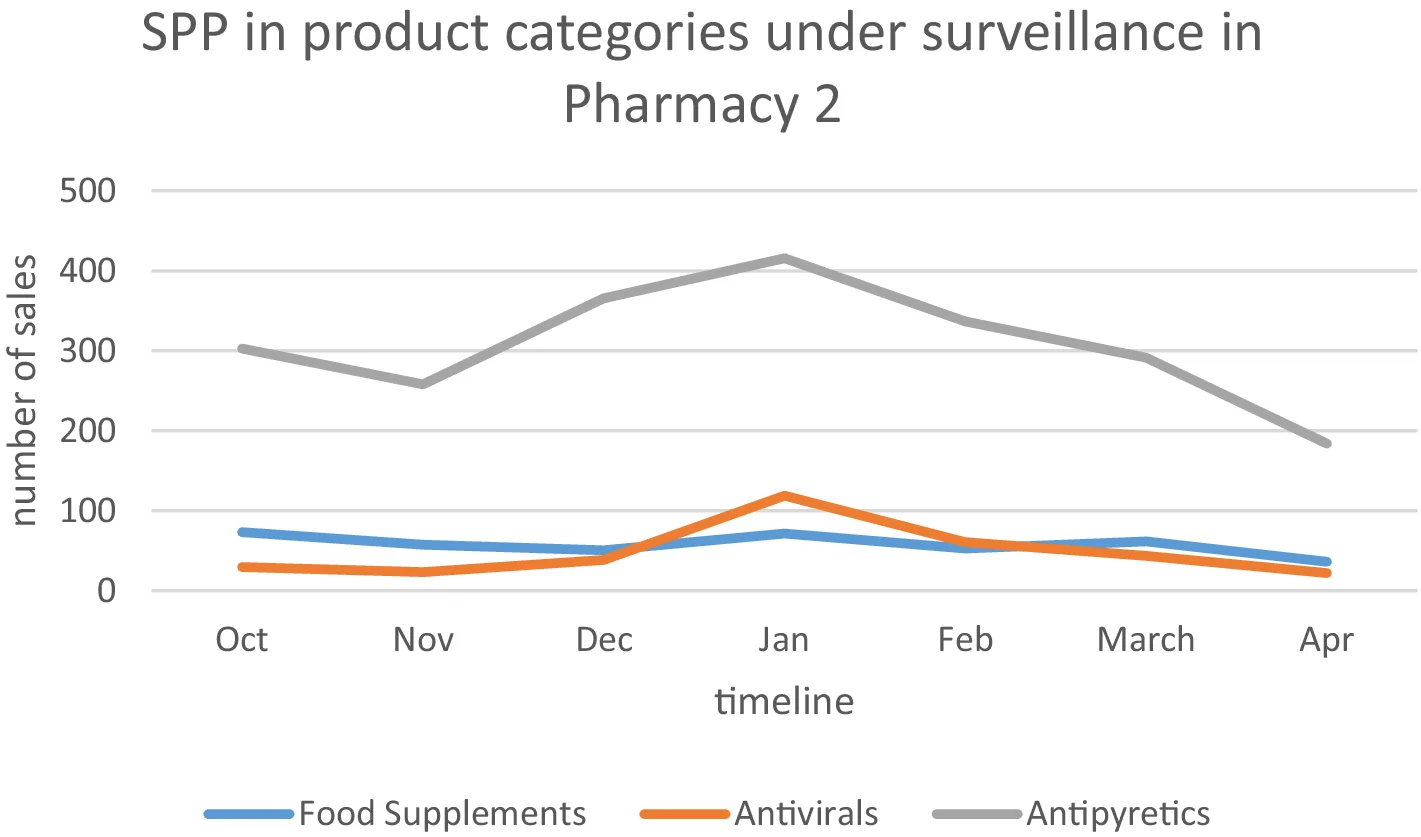
Distribution of sales across the monitored product categories in Pharmacy 2, October 2024–April 2025.

### 3.2 Temporal variation in pharmacy sales

Mean SPP vary from 121.06 units in the category of antivirals to 685.96 units in the group of antipyretics. The greatest range of variety between monthly sales within each category is observed for the antipyretics (*R* = 493.07, CV = 22.8, SD = 3.26%) and antivirals (*R* = 216.4, CV = 64.78, SD = 9.25%). Moderate fluctuations are registered for the supplements group (*R* = 160.16, CV = 33.23, SD = 4.75%) and the products for nasal congestion/rhinorrhea relief (*R* = 156, CV = 15.79, SD = 2.26%). Sales of products for sore throat show relative stability (*R* = 103, CV = 18.62, SD = 2.66%) without substantial informative value for surveillance.

The highest monthly proportions of antipyretic and antiviral sales were recorded in January 2025. January accounted for 19.14% of total antipyretic sales and 32.00% of total antiviral sales during the 7-month observation period. The proportion of antipyretics sold in January was similar in Pharmacy 1 and Pharmacy 2—19.01 and 19.30%, respectively. For products marketed as antivirals, the corresponding proportions were 29.80% in Pharmacy 1 and 35.36% in Pharmacy 2. Food supplement sales also reached one of their highest monthly levels in January, accounting for 19.66% of total sales in this category. However, a nearly identical proportion was observed in October 2024 (19.65%), indicating that the January pattern was not specific to all monitored categories (Figure 3).

**Figure 3:**
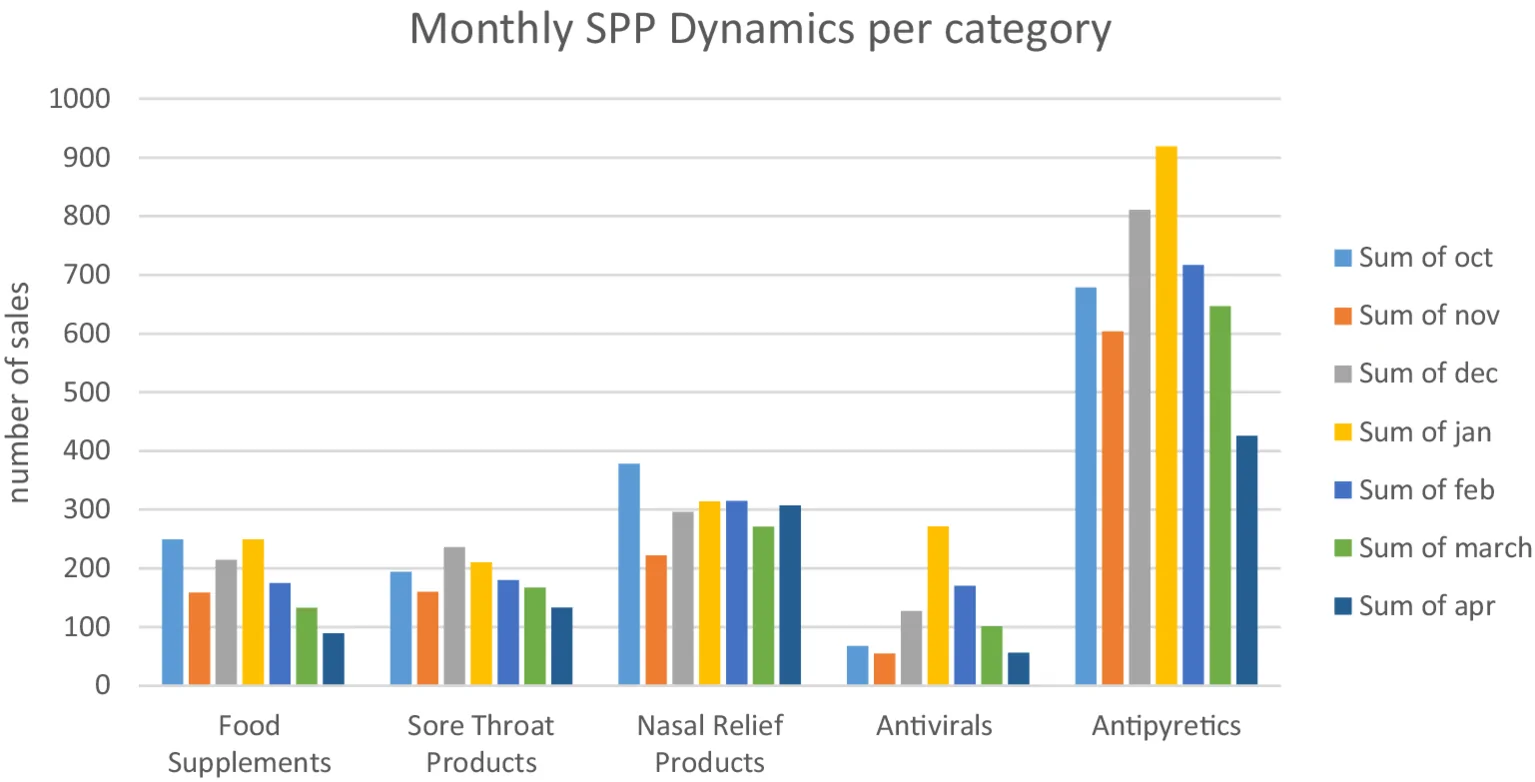
Monthly distribution of sales by monitored product category, October 2024–April 2025.

The Friedman test indicated a statistically significant difference in monthly sales distributions across products (*p* < 0.05). Mean rank values suggest that January had the highest relative sales, while April had the lowest.

Post-hoc comparisons between January and each of the other 6 months were performed using Wilcоxon signed-rank tests with a Bonferroni correction of *α* = 0.0083. Although sales in January were generally higher than those in the other months, none of the pairwise comparisons reached the adjusted level of statistical significance. Several comparisons produced unadjusted *p*-values of approximately 0.012, but these findings were not statistically significant after correction for multiple testing. The January increase should therefore be interpreted as a descriptive temporal pattern rather than a statistically confirmed peak.

## 4 Discussion

This pilot study demonstrated the feasibility of retrospectively collecting and analysing aggregated monthly sales data for selected over-the-counter respiratory products from two community pharmacies in Sofia. Antipyretic and antiviral product sales were highest in January 2025, broadly coinciding with the reported seasonal peak in acute respiratory infections (ARI) in Sofia during week 4 of 2025 (34). However, none of the post-hoc comparisons between January and the other months remained statistically significant after Bonferroni correction. The findings should therefore be interpreted as descriptive temporal concordance rather than evidence of a statistically confirmed association, predictive performance, or validated surveillance capability.

The observed patterns are broadly consistent with previous studies reporting associations between pharmacy sales and respiratory infection activity. International research has identified correlations between sales of selected pharmaceutical products and influenza-like illness, although the strength and consistency of these associations vary by product category and setting (15, 16). Studies from Canada have also identified antiviral dispensing as a potentially useful indicator of influenza activity (35). Nevertheless, the present results cannot be directly compared with these studies because of differences in product classification, data frequency, healthcare systems, OTC availability, and health-seeking behaviour.

A similar study carried out in the USA, Pennsylvania, that retrieved sales data on fever, cough and cold and antidiarrheal products between 2016 and 2021 supports the utility of SPP information for public health surveillance as well. The authors report that the highest daily sales occurred in the cough and cold category, while our results demonstrate a higher demand for antipyretics. We interpret the difference through the grouping approach, as the US study analysed together medications for nasal, throat, and cough relief (36).

Older data from 2013 from the same area suggest dynamics of OTC demands for upper and lower respiratory symptom treatment together with thermometer purchases are significantly associated with patient volume during influenza season despite the time lag of two and one days (24). The utility of SPP data in the US context has been also highlighted in a report produced by the New York City Department of Health and Mental Hygiene, which confirms observations of influenza-like illness-related OTC sales being highest during annual influenza epidemics (28).

Multiple factors, apart from the incidence of respiratory infections, influence pharmacy sales. These include television advertising and promotional campaigns, price changes, seasonal purchasing behaviour, health literacy, and consumer preferences (33, 37). Prescribing and dispensing practices, medicine availability or shortages, stockpiling, and healthcare accessibility may introduce additional variation (32, 35). Sales may also be affected by environmental exposures such as air pollution (38) and by non-infectious respiratory conditions, including allergies, asthma, and chronic obstructive pulmonary disease (39). Population mobility may introduce additional variation (40). These factors reduce the epidemiological specificity of pharmacy-derived indicators and may generate increases or decreases in sales independently of infectious disease activity.

Despite these limitations, pharmacy sales data may have value as one component of a broader syndromic surveillance framework. Such data are routinely generated and could potentially complement clinical, laboratory, absenteeism, and other behavioural data streams (4, 6). Their usefulness is likely to depend on standardized product classification, consistent participation by a sufficiently representative pharmacy network, automated and frequent data transfer, and validation against established surveillance indicators. The present study should therefore be regarded as an initial feasibility assessment that demonstrates the practicality of collecting pharmacy sales information rather than as validation of an operational early-warning indicator.

### 4.1 Limitations

This study has several important limitations. First, sales data were obtained from only two community pharmacies in Sofia during a single respiratory season. The participating pharmacies cannot be considered representative of Sofia or Bulgaria, and the findings have limited external validity. Second, the retrospective design and small number of observations restricted the statistical analysis and prevented prospective assessment of surveillance performance.

Third, product selection was partly based on television advertising observed during February–March 2025, although the sales period began in October 2024. This temporal mismatch may have introduced retrospective and commercial selection bias. Consultation with pharmacists helped refine and broaden the initial product list but could not eliminate this potential bias. Furthermore, the operational product categories were not based on a formal ATC classification, which may limit reproducibility and comparability with other studies.

Fourth, the available product categories differed between the two pharmacies, and information on potential determinants of sales—such as stock availability, shortages, prices, promotional activities, customer characteristics, prescriptions, and clinical diagnoses—was unavailable. Finally, monthly aggregation prevented formal time-series, correlation, and lag analyses. Consequently, the study cannot establish an association between pharmacy sales and ARI incidence or determine whether changes in sales preceded routine surveillance signals. Larger prospective multicentre studies using standardized classifications, daily or weekly sales data, and appropriate epidemiological comparison series are required.

## 5 Conclusion

This pilot study demonstrated the feasibility of collecting and analysing aggregated monthly sales data for selected over-the-counter respiratory products from two community pharmacies in Sofia. Antipyretic and antiviral sales were highest in January 2025, broadly coinciding with the reported seasonal peak in acute respiratory infections. Although previous international studies suggest that pharmacy sales may precede some conventional surveillance signals, the limited sample and monthly aggregation of the present data did not allow assessment of correlation, predictive performance, or lead time.

If validated through larger prospective studies, pharmacy sales data could complement existing surveillance systems and potentially support earlier situational awareness, healthcare resource allocation, hospital preparedness, and public health planning. Combining these data with established epidemiological, clinical, laboratory, and environmental indicators may improve the accuracy and reliability of future surveillance models.

Future research should involve representative multicentre pharmacy networks, standardized product classifications, and daily or weekly sales data. Continued collaboration among public health authorities, researchers, community pharmacists, and data scientists will be essential to determine whether pharmacy sales can contribute to more responsive and resilient surveillance systems.

## Data Availability

The raw data supporting the conclusions of this article will be made available by the authors, without undue reservation.
